# Gynecologic Malignancies in Obstructed Hemivagina and Ipsilateral Renal Anomaly (OHVIRA) Syndrome: A Systematic Review

**DOI:** 10.3390/jcm15082824

**Published:** 2026-04-08

**Authors:** Giuseppe Parisi, Emanuele Perrone, Ilaria Capasso, Matteo Bruno, Maria Consiglia Giuliano, Nicola Macellari, Marco D’Indinosante, Francesco Fanfani

**Affiliations:** Unità Operativa Complessa di Ginecologia Oncologica, Dipartimento Scienze della Salute della Donna, del Bambino e di Sanità Pubblica, Fondazione Policlinico Universitario A. Gemelli, IRCCS, Largo Agostino Gemelli, 8, 00136 Roma, Italy; giuseppe.parisi01@icatt.it (G.P.); emanuele.perrone@policlinicogemelli.it (E.P.); ilaria.capasso1@guest.policlinicogemelli.it (I.C.); matteo.bruno@guest.policlinicogemelli.it (M.B.); mariaconsiglia.giuliano01@icatt.it (M.C.G.); nicola.macellari01@icatt.it (N.M.); francesco.fanfani@policlinicogemelli.it (F.F.)

**Keywords:** OHVIRA, Herlyn–Werner–Wunderlich syndrome, Müllerian anomaly, gynaecologic cancer, fertility-sparing surgery

## Abstract

**Background:** Obstructed hemivagina and ipsilateral renal anomaly (OHVIRA) syndrome, also known as Herlyn–Werner–Wunderlich syndrome (HWWS), is a rare Müllerian malformation. Gynaecologic malignancies reported in association with OHVIRA syndrome/HWWS are exceptional and scattered across isolated case reports and small case series, leading to significant challenges for screening, early diagnosis, and optimal management. The primary aim of this study was to comprehensively review and synthesize the clinicopathologic features, treatment approaches, and reported outcomes of patients with OHVIRA-associated gynaecologic neoplasms. **Methods:** A systematic review of published cases of gynecologic malignancies in OHVIRA/HWWS was conducted using PubMed/MEDLINE and Scopus from database inception through January 2026. To expand the current evidence, an illustrative novel institutional case is also presented and integrated into the analysis. **Results:** A total of 21 cases were identified and analysed; reported tumours predominantly involved the lower genital tract (vagina/cervix), with a recurrent representation of adenocarcinoma, particularly clear cell histology, and a frequent origin from the obstructed or non-visible compartment when side was described. Endometrial and ovarian involvement was uncommon. **Conclusions:** The anatomical complexity of OHVIRA syndrome creates a diagnostic blind spot that warrants heightened clinical suspicion, rigorous MRI-based anatomic delineation, and side-specific evaluation in symptomatic patients. By synthesizing the available literature, this review underscores the necessity for tailored, multidisciplinary management and proactive surveillance strategies in this rare population.

## 1. Introduction

Obstructed hemivagina and ipsilateral renal anomaly (OHVIRA) syndrome, also referred to as Herlyn–Werner–Wunderlich syndrome (HWWS), is defined by uterus didelphys (or bicorporal uterus), obstructed hemivagina, and ipsilateral renal anomaly, most commonly renal agenesis [1]. It represents a rare congenital anomaly resulting from combined developmental defects of the paramesonephric (Müllerian) and mesonephric (Wolffian) ducts [1]. Wolffian duct anomalies may impair both renal formation and Müllerian development: the Wolffian ducts contribute to renal development and induce normal Müllerian tract development, and their abnormal development has been linked to unilateral renal agenesis with an imperforated hemivagina. An additional embryologic framework (Acién’s hypothesis) proposes a Wolffian contribution to vaginal development, whereby developmental arrest of the homolateral mesonephros/mesonephric duct may lead simultaneously to absent ureter/kidney, blind hemivagina, and failure of Müllerian fusion with resultant uterus didelphys [2].

Müllerian duct anomalies have been estimated to have a prevalence of 5–7% among women [3]. Within this broader spectrum, obstructive Müllerian anomalies estimated occurrence is 0.1–3.8% [4,5].

The ESHRE/ESGE consensus on the classification of female genital tract congenital anomalies defines this uterine malformation as U3b-C2-V2, corresponding to a complete bicorporal uterus with double “normal” cervix and longitudinal obstructing vaginal septum [6].

Clinically, OHVIRA syndrome is most often recognized after menarche, when menstrual outflow obstruction leads to progressive symptoms. In a recent systematic review and meta-analysis, the most common presenting symptoms were abdominal pain and dysmenorrhea, reported in 67% and 64% of cases, respectively, while hematocolpos and hematometra were among the most frequent associated findings, occurring in 55% and 53% of cases [7].

Beyond adolescence, HWWS can also be identified in the prepubertal period, during urologic evaluation of an absent/dysplastic kidney detected on prenatal or early-life ultrasonography [8]. Zhu et al. proposed a clinically useful classification based on complete versus incomplete hemivaginal obstruction, which helps explain why some patients present shortly after menarche with obstructive/cyclic symptoms (class 1), whereas those with incomplete vaginal obstruction and partial drainage (class 2), may present later with less specific manifestations such as purulent/bloody vaginal discharge and ascending genital tract infection years after menarche [1].

Early recognition and surgical correction of the obstructing vaginal septum are recommended to prevent hematocolpos/hematometra, endometriosis, infection, and impaired fertility. Therefore, early diagnosis and surgical treatment are crucial to prevent complications; vaginal septum resection remains the gold-standard treatment, with hysteroscopic septum resection representing a promising minimally invasive alternative [7,9].

However, while the management of benign complications is well-established, the development of malignant complications in the context of OHVIRA syndrome represents an exceptional and poorly understood clinical entity. Rare gynaecologic malignancies have been sporadically described in OHVIRA syndrome, mostly involving the cervix and vagina. The exceptionally low case numbers and heterogeneous reporting in the current literature have limited the ability to recognize patterns of presentation, risk factors, and outcomes. Therefore, the primary aim of this study is to comprehensively review and synthesize the clinicopathologic features, risk factors, treatment approaches, and outcomes of patients with OHVIRA-associated gynaecologic neoplasms, integrating a novel illustrative index case to expand the available evidence.

## 2. Materials and Methods

A systematic review of the literature was conducted to identify published reports of gynecologic malignancies and borderline ovarian tumors occurring in patients with OHVIRA syndrome (Herlyn–Werner–Wunderlich syndrome).

### 2.1. Search Strategy

**Figure 1:**
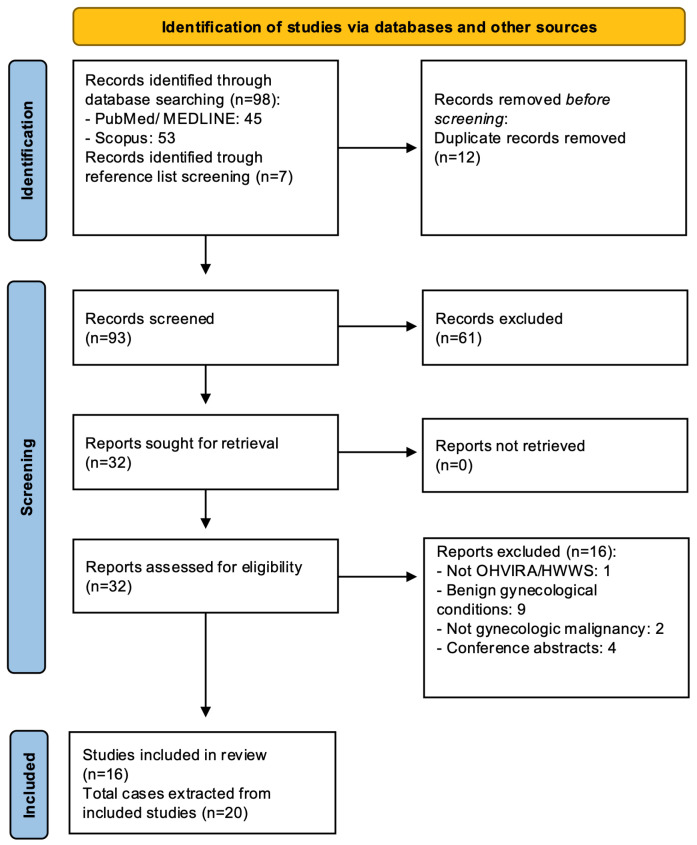
PRISMA 2020 flow diagram of study selection [10].

This systematic review was conducted and reported in accordance with the PRISMA 2020 statement (Preferred Reporting Items for Systematic Reviews and Meta-Analyses) [10]. The PRISMA 2020 checklist is provided in Supplementary Table S1. This systematic review was not prospectively registered, and no formal protocol was developed. A literature search was performed in PubMed/MEDLINE and Scopus from database inception through January 2026, using the terms “OHVIRA”, “Herlyn–Werner–Wunderlich syndrome”, and related combinations with broader gynecologic oncology keywords referring to neoplastic conditions of the cervix, vagina, endometrium, and ovary. To maximize case retrieval, the reference lists of included articles were manually reviewed for additional eligible reports. The study selection process is summarized in Figure 1.

### 2.2. Study Selection

All retrieved records were imported into Rayyan (Qatar Computing Research Institute, Doha, Qatar), where duplicate records were removed before screening [11]. Title and abstract screening were independently performed by two reviewers (GP and MCG). Discrepancies were resolved by consultation with a third reviewer (MD). Potential duplicate case reporting across publications was assessed by cross-checking authorship, year of publication, patient age, tumor site, and key clinical details. Titles and abstracts were screened for relevance, and eligible articles underwent full-text evaluation.

Inclusion criteria were: articles published in peer-reviewed scientific journals; articles describing gynecologic malignancies in patients with confirmed OHVIRA syndrome/HWWS; and articles published in English. Exclusion criteria were: non-English language publications; non-clinical or experimental studies; articles lacking sufficient clinical or surgical detail. Conference abstracts, editorials, letters, and unpublished reports were excluded.

### 2.3. Data Synthesis

Given the rarity of the condition, the limited number of reported cases, and the heterogeneity of the available literature, a quantitative synthesis was not feasible. Therefore, findings were summarized using a qualitative descriptive approach and grouped into two main domains: clinicopathologic features of the reported gynecologic malignancies and the corresponding obstetric history, treatment strategies, and oncologic outcomes. Because the available evidence was predominantly case-based, formal risk-of-bias assessment using conventional tools was not applicable. Instead, the methodological quality of the included reports was evaluated qualitatively, focusing on key domains such as diagnostic clarity, completeness of the clinical and pathological description, and adequacy of reported oncologic outcomes.

To expand the available evidence, an illustrative novel case managed at our tertiary center was additionally included. Written informed consent was obtained from the patient for the publication of clinical details and images related to the case.

## 3. Results

### 3.1. Illustrative Case

#### 3.1.1. Clinical Presentation

A 22-year-old woman with a history of OHVIRA syndrome presented with bilateral adnexal masses incidentally detected during routine gynaecologic ultrasound.

#### 3.1.2. Imaging Findings

**Figure 2:**
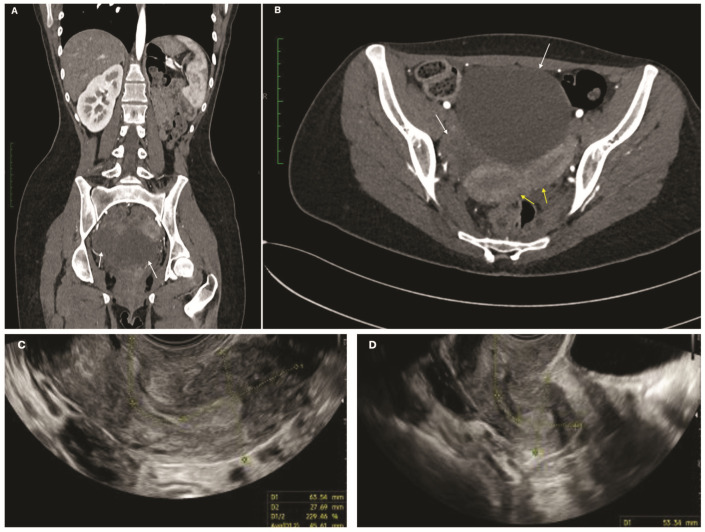
Preoperative imaging of the illustrative institutional case. (**A**,**B**) Preoperative CT showing uterus didelphys (yellow arrows), left renal agenesis, and bilateral adnexal masses (white arrows). (**A**) Coronal view. (**B**) Axial pelvic view. (**C**,**D**) Transvaginal ultrasound showing a bicorporal uterus (U3b/C2) with two distinct endometrial cavities.

Transvaginal ultrasound revealed a right unilocular solid mass (71 × 31 mm) and a left cystic-solid lesion (35 × 32 mm). Serum CA-125 was 51.5 U/mL. Contrast-enhanced computed tomography confirmed uterus didelphys, absence of the left kidney, and bilateral adnexal masses with minimal free fluid (Figure 2A,B).

#### 3.1.3. Initial Surgery and Pathology

The first laparoscopic procedure was performed at a secondary-level hospital and included peritoneal washings, bilateral ovarian cystectomy, omental biopsy, and multiple peritoneal biopsies. Ovarian cyst spillage occurred during cystectomy; both cysts were retrieved in an endoscopic bag.

Histopathology demonstrated bilateral serous borderline ovarian tumour with focal microinvasion and non-invasive peritoneal implants; peritoneal washings were positive for neoplastic cells. The disease was staged as pT3a (AJCC), FIGO IIIA2.

#### 3.1.4. Multidisciplinary Management and Restaging

The patient was referred to a tertiary gynaecologic oncology centre. After multidisciplinary tumour board review, fertility-sparing laparoscopic restaging with infracolic omentectomy and excision of a residual right ovarian lesion was recommended, together with referral to an oncofertility service.

Pre-restaging ultrasound by expert operators confirmed uterus didelphys (U3bC2). (Figure 2C,D).

Laparoscopic infracolic omentectomy and enucleation of the residual right ovarian lesion were performed.). No additional macroscopic disease was observed. The postoperative course was uneventful and the patient was discharged on postoperative day 2.

#### 3.1.5. Follow-Up

The patient underwent oocyte cryopreservation after oncofertility counselling. She is currently followed every 3 months with pelvic ultrasound and CA-125. At early follow-up (6 months from initial diagnosis; 3 months from restaging), she showed no evidence of disease.

This illustrative case expands the extremely limited literature describing ovarian tumours in OHVIRA/HWWS and highlights the feasibility of fertility-sparing management in carefully selected young patients.

### 3.2. Results of the Literature Review

A total of 16 articles reporting 20 cases of gynecologic malignancy or borderline tumor occurring in the setting of OHVIRA/HWWS were identified; together with the present case, 21 cases were included in the descriptive analysis. The clinicopathologic characteristics of the reported cases are summarized in Table 1.

Peer-reviewed published cases of OHVIRA-associated malignancies consist primarily of isolated case reports and small case series; three narrative reviews were included [10,20,21]. When reported (20/21; 95%), patient age ranged from 20 to 74 years, with a mean age of 41 years. The most frequent primary tumour site was cervix (12/21, 57.1%), followed by vagina (5/21, 23.8%), endometrium (2/21, 9.5%), and ovary (2/21, 9.5%). Most cervico-vaginal tumours were reported on the obstructed side when laterality was provided (13/16, 81.3%).

Histologically, adenocarcinoma histotype predominated among cases. Clear cell adenocarcinoma represented the single most recurrent subtype, accounting for 9/21 cases (42.9%), including 5 cervical and 4 vaginal clear cell carcinomas. Endometrioid adenocarcinoma was reported in 3/21 cases (14.3%) (two cervical and one endometrial), while serous adenocarcinoma of the endometrium was described in 1/21 case (4.8%). Borderline ovarian tumours comprised 2/21 cases (9.5%) (AlMulhim et al. [20] and our illustrative case). 

HPV status was infrequently reported; among cases with available data, HPV was positive in only 1 case (1/4, 25%) with a positivity for HPV 16. Diethylstilbestrol (DES) in utero exposure was consistently absent when reported (10/10).

Obstetric history, treatment strategies, and oncologic outcomes are summarized in Table 2.

Obstetric history was reported in 15 cases, including 6 nulligravid (40%), 1 primiparous (6.7%), and 8 multiparous (53.3%) patients; obstetric history was not reported in the remaining 6 cases. No consistent obstetric pattern emerged.

Disease stage at diagnosis was heterogeneous. Among cervical cancers, 9 of 12 cases (75%) were diagnosed at early stages (FIGO IA1-IIA1, excluding IB3), while the remaining 3 of 12 cases (25%) presented with locally advanced disease (FIGO IB3-IVA). No cases with distant metastatic disease (FIGO IVB) were reported. Vaginal cancers were predominantly reported as FIGO I (4/5; 80%), with only one FIGO III case (1/5; 20%). Among endometrial cancers, one case (50%) was classified as FIGO 2009 stage IIIA and one (50%) as stage IIIC1. Borderline ovarian tumors were reported as FIGO stage IA (1/2; 50%) and stage IIIA2 (1/2; 50%).

Treatment approaches reflected the primary tumour site and stage. The majority of cervico-vaginal malignancies were managed surgically with radical hysterectomy (simple or radical), often combined with vaginectomy and nodal assessment (PLND ± PALND). Multimodality therapy was common in locally advanced disease, including chemoradiotherapy and, in selected cases, anterior pelvic exenteration. Endometrial cancers were managed with surgical staging and adjuvant therapy as reported. Borderline ovarian tumours were in both cases managed conservatively with fertility-sparing surgery.

Follow-up duration was available for 16 of 21 cases (76%) and ranged from 3 to 60 months, with a mean follow-up of 21 months. Among these cases, 13 patients (81%) were reported as having no evidence of disease (NED), whereas 3 patients (19%) died of disease (DOD); all deaths occurred in cervical cancer cases from the Zong et al. series [20].

## 4. Discussion

### 4.1. Discussion of Main Findings

#### 4.1.1. Tumour Spectrum and Histologic Profile

In this case-based synthesis (20 published cases plus the present case; *n* = 21), malignancies overwhelmingly involved the lower genital tract, with the cervix and vagina accounting for 16/21 cases (76.2%), whereas endometrial (2/21, 9.5%) and ovarian (2/21, 9.5%) involvement was uncommon.

A striking feature of the published OHVIRA/HWWS malignancy literature is the predominance of adenocarcinoma, with clear cell adenocarcinoma representing the single most recurrent subtype in our dataset (9/21, 42.9%), spanning both cervix and vagina. Mabuchi et al. similarly emphasized the relative over-representation of clear cell carcinoma among HWWS-associated gynaecologic cancer [12]. This “clear cell signal” is clinically meaningful because clear cell cervico-vaginal tumours are often HPV-independent, may not be captured by routine HPV-based screening paradigms, and can present with non-specific bleeding even when the visible cervix appears normal.

One plausible pathway is persistent adenosis/metaplastic glandular epithelium in the septum or obstructed segment, potentially exposed to chronic inflammation or haemorrhagic products. Another hypothesis is long-standing occult endometriosis or retrograde menstruation in obstructed compartments, although direct mechanistic evidence is limited.

Notably, the historical paradigm linking cervico-vaginal clear cell carcinoma to prenatal DES exposure does not appear to explain many OHVIRA-related cases, as discussed below.

#### 4.1.2. Lateralization and the “Occult Compartment” Problem

**Figure 3:**
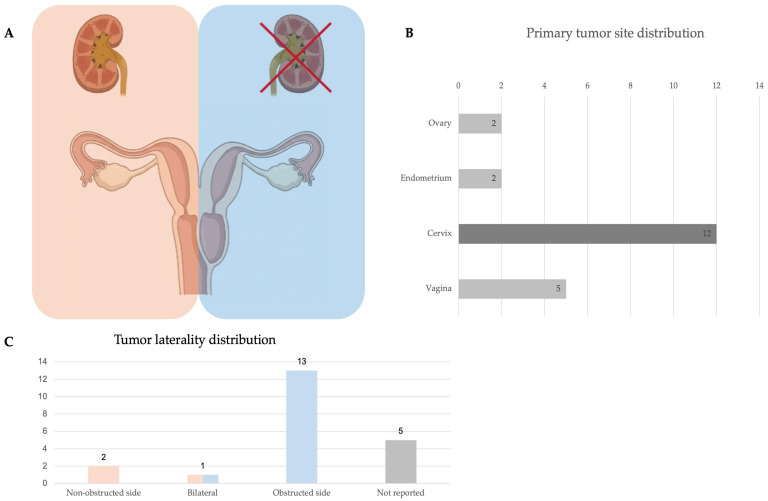
Composite visual summary of the main clinicopathologic patterns reported in OHVIRA/HWWS-associated gynecologic malignancies. (**A**) Schematic representation of the anatomical hallmarks of OHVIRA syndrome: uterus didelphys, obstructed hemivagina and ipsilateral renal agenesis. (**B**) Distribution of primary tumor site across reported cases. (**C**) Distribution of tumor laterality across reported cases.

Where laterality was reported, tumours frequently arose on the obstructed side (81.3%), supporting the practical concept that the obstructed/non-visible hemicervix or hemivagina functions as an occult compartment where lesions may develop and remain clinically silent or diagnostically inaccessible until symptomatic. A composite visual summary integrating the distribution of primary tumor site and tumor laterality across reported cases within the anatomic framework of OHVIRA/HWWS is provided in Figure 3.

Lei and Zhang’s review of cervico-vaginal carcinomas in HWW syndrome specifically noted that the vast majority of tumours occurred on the obstructed side when the side was available, and argued that this contributes to delayed detection because these lesions may only become visible after septum resection or through dedicated imaging [23].

From a mechanistic standpoint, chronic obstruction with retained secretions/hematocolpos and recurrent inflammation is frequently invoked as a plausible carcinogenic co-factor. Mabuchi et al. discuss a biologically plausible pathway involving iron-rich retained menstrual blood and oxidative stress (reactive oxygen species), conceptually paralleling endometriosis-associated carcinogenesis; nonetheless, they explicitly acknowledge that direct evidence is lacking [12]. Accordingly, the most defensible interpretation is not proven causality, but rather that anatomy-driven diagnostic vulnerability and chronic inflammatory microenvironment could both contribute. These proposed mechanisms remain biologically plausible but unproven, and should therefore be considered hypothesis-generating rather than representative of an established pathogenic mechanism.

#### 4.1.3. HPV Status and DES Exposure

Across our compiled cases, HPV testing was infrequently reported, with most entries listed as “not reported”. When available, HPV was mostly negative [13,17]; only Lei & Zhang reported HPV16 positivity in their vaginal clear cell case [21]. These findings suggest that not all cervico-vaginal cancers in OHVIRA follow classic HPV-driven pathways and that HPV-negative glandular malignancies arising from a non-visible cervix should be considered in the differential diagnosis. In this setting, reliance solely on cytology or HPV testing of the visible cervix may fail to detect lesions originating from the obstructed or non-visible compartment.

Regarding DES exposure, the available evidence is more consistent: when explicitly reported, prenatal DES exposure was absent in all cases. This observation is particularly relevant given the well-established historical association between in utero DES exposure, vaginal or cervical adenosis, and subsequent development of clear cell carcinoma [28,29]. It suggests that clear cell histology can occur without DES, possibly through alternative pathways (e.g., congenital adenosis/metaplasia and chronic local factors).

#### 4.1.4. Stage at Presentation and Symptom Profile

The reported stage distribution was heterogeneous, ranging from early-stage to advanced disease. This heterogeneity is consistent with a dual reality: some patients are diagnosed promptly (especially those previously known to have OHVIRA/HWWS and followed more closely), while others present late because symptoms overlap with benign OHVIRA manifestations or because the lesion resides in an inaccessible compartment. Mabuchi et al. describe precisely this diagnostic challenge showing that patients may have a normal Pap smear from the visible cervix with malignancy arising in the remnant obstructed compartment [12].

Early diagnosis of OHVIRA/HWWS is important not only to facilitate timely management of the anomaly itself but also to reduce the risk of avoidable short- and long-term complications. In the recent systematic review and meta-analysis by Bonetti et al., the pooled mean age at symptom onset was 14.45 years, whereas the pooled mean age at diagnosis was 16.36 years, suggesting a clinically meaningful diagnostic delay [7].

Accordingly, early recognition of the anomaly and timely surgical correction of the obstructing vaginal septum are essential not only to relieve symptoms but also to reduce the risk of progressive morbidity and delayed gynaecologic evaluation. In the setting of OHVIRA-associated malignancies, this principle may be even more relevant because failure to recognize the underlying anatomy early may further contribute to the delayed detection of lesions arising in the concealed compartment.

#### 4.1.5. Reproductive and Obstetric Background

Obstetric history was inconsistently reported and did not reveal a recognizable reproductive profile. The available data therefore do not support any inference regarding parity or prior pregnancy history as clinically informative features in OHVIRA-associated gynecologic malignancies. More broadly, this appears consistent with OHVIRA literature, where successful pregnancies have been described, suggesting that reproductive potential may be preserved in selected and appropriately managed patients [30].

#### 4.1.6. Treatment Patterns and Oncologic Outcomes

Management in the reported cases largely followed oncologic standards: radical surgery and/or chemoradiotherapy, yet OHVIRA/HWWS anatomy introduces unique practical barriers. Lei & Zhang highlight that anatomic distortion can complicate radiotherapy delivery (including brachytherapy applicator placement), and they recommend individualized planning, often under anaesthesia, and MRI-led follow-up where anatomy precludes direct visualization [23].

Oncologic outcomes are difficult to interpret because follow-up is variably reported, and many cases have not reported outcomes. Nonetheless, among cases with documented endpoints, NED is frequent, while DOD events, clustered within the Zong series, reported several advanced adenocarcinomas with poor outcomes [22]. This reinforces an intuitive point: prognosis appears more closely tied to disease stage and feasibility of adequate treatment than to OHVIRA/HWWS per se.

#### 4.1.7. Role of Serum Markers

In obstructive Müllerian anomalies, elevated serum markers such as CA-125 may reflect benign obstruction-related conditions rather than an underlying neoplastic process. Hematocolpos, hematometra, retrograde menstruation, secondary endometriosis, and peritoneal irritation may all contribute to increased circulating marker levels, potentially raising suspicion for malignancy. However, given their limited specificity in this context, they should not be regarded as reliable diagnostic or prognostic indicators. Rather, they should be considered ancillary findings and always interpreted in conjunction with clinical presentation, anatomic assessment, and imaging findings [31,32].

#### 4.1.8. Ovarian Borderline Tumours in OHVIRA/HWWS

Ovarian involvement was exceedingly rare in the published OHVIRA/HWWS malignancy literature: beyond the present patient, we identified only a single previously reported ovarian case, and it was likewise a serous borderline ovarian tumour (BOT) [20]. The present report therefore expands the phenotype by documenting BOT with higher-risk features: focal microinvasion, positive washings, and non-invasive peritoneal implants (FIGO IIIA2), managed with fertility intent.

Fertility-sparing surgery can be appropriate in carefully selected patients, but recurrence risk rises with bilaterality, microinvasion, and implants, making staging quality, counselling, and follow-up rigorous [33,34]. In OHVIRA patients, an additional layer is reproductive planning in the context of Müllerian duplication/obstruction and potential need for corrective procedures; therefore, integrating oncofertility referral and long-term surveillance planning is particularly critical [35].

### 4.2. Strengths and Weaknesses

To our knowledge, this review represents the most comprehensive synthesis to date of gynecologic malignancies reported in association with OHVIRA/HWWS, consolidating all currently available cases (20 from peer-reviewed literature plus the present case) into a structured dataset. By expanding the available case-based evidence, our compilation highlights clinically actionable signals, including the predominance of lower genital tract involvement, the recurrent clear cell histology, and the frequent occurrence on the obstructed/non-visible side, all of which carry direct implications for diagnostic work-up and multidisciplinary planning.

Nevertheless, several limitations should be acknowledged. First, this review is based predominantly on isolated case reports and small case series, with the inherent risk of publication and selection bias. Second, reporting across the included articles was highly heterogeneous, with frequent missing data regarding HPV status, DES exposure, tumor markers, staging details, treatment, and oncologic outcomes. Third, the very limited number of available cases precludes any reliable estimation of incidence, causal inference, or meaningful comparison across tumor types and management strategies. Fourth, follow-up duration was often short, precluding any meaningful assessment of long-term oncologic outcomes. Finally, the currently available data do not allow any meaningful comparison of the incidence of gynecologic malignancies between patients with OHVIRA/HWWS and the general population.

### 4.3. Implications for Clinical Practice and Future Research

Taken together, the literature suggests a concrete, practice-oriented message: OHVIRA/HWWS creates a screening and diagnostic “blind spot.” Symptoms such as irregular bleeding, postcoital bleeding, or unilateral pelvic pain should prompt side-specific evaluation rather than routine “single-cervix” workflows. When direct inspection of the obstructed hemivagina/hemicervix is not possible, MRI-based assessment becomes the most reliable tool for anatomic delineation and lesion detection, providing superior soft-tissue contrast and anatomic delineation in Müllerian anomalies. Where feasible, early septum resection not only relieves symptoms but also enables direct visualization and biopsy of the previously concealed compartment. In most published cases, oncologic treatment followed standard site-specific guidelines (radical surgery and/or chemoradiation), but anatomical distortion can complicate applicator geometry for brachytherapy, target delineation, and surgical planes.

Therefore, careful multidisciplinary planning involving gynecologic oncology, radiology, radiation oncology, and urology is essential to optimize oncologic management in this non-standard anatomy, particularly given the potential challenges in surgical planning, brachytherapy delivery, and the possible coexistence of additional genitourinary malformations that may influence diagnostic work-up and long-term surveillance [36,37].

The field would benefit from multicentre registries that systematically capture OHVIRA/HWWS phenotype, prior corrective surgery, imaging surveillance, HPV status, adenosis/endometriosis markers, and standardized oncologic outcomes evaluation.

## 5. Conclusions

Gynecologic malignancies reported in association with OHVIRA/HWWS are rare but appear to involve the lower genital tract predominantly, with a recurrent representation of adenocarcinoma, particularly clear cell histology, and a frequent origin from the obstructed or non-visible compartment when laterality is described. This anatomic “blind spot” may contribute to delayed recognition, underscoring the need for heightened clinical suspicion and side-specific evaluation in symptomatic patients, supported by MRI-based anatomic delineation when direct visualization is limited. Although causality and true incidence cannot be inferred from case-based evidence, consolidating these reports provides practical insights for diagnosis, multidisciplinary planning, and long-term surveillance, and highlights the need for standardized reporting and multicenter registries.

## Figures and Tables

**Table 1:** Clinicopathologic characteristics of reported gynaecologic malignancies in OHVIRA/HWWS syndrome.

Author	Age	Cancer	Histology	Site	HPV Status	DES Exposure
Mabuchi et al. 2022 [12]	74	Cervical cancer (FIGO IIA1)	Clear cell adenocarcinoma	Obstructed side	NR	No
Watanabe et al. 2012 [13]	33	Cervical cancer (FIGO IVA)	Endometrioid adenocarcinoma	Obstructed side	Neg	No
	53	Vaginal cancer (FIGO I)	Clear cell adenocarcinoma	Obstructed side	Neg	No
Kaba et al. 2013 [14]	49	Cervical cancer (FIGO IB1)	Endometrioid adenocarcinoma	Obstructed side	NR	NR
Cordoba et al. 2017 [15]	37	Cervical cancer (FIGO IIIA)	Adenocarcinoma	Obstructed side	NR	NR
Kusunoki et al. 2018 [16]	65	Cervical cancer (FIGO IB2)	Clear cell adenocarcinoma	Obstructed side	NR	No
Oka et al. 2020 [17]	38	Cervical cancer (FIGO IIA1)	Adenocarcinoma	Obstructed side	Neg	No
Mei et al. 2020 [18]	40	Vaginal cancer (FIGO I)	Clear cell adenocarcinoma	Obstructed side	NR	No
Tanase et al. 2021 [19]	52	Cervical cancer (FIGO IB1)	Clear cell adenocarcinoma	Obstructed side	NR	No
Almulhim et al. 2021 [20]	29	Borderline Ovarian Tumor (FIGO IA)	Serous borderline tumor	Obstructed side (ovary)	NA	NA
Kobayashi et al. 2021 [21]	58	Endometrial cancer (FIGO 2009 IIIA)	Endometrioid adenocarcinoma	Non-obstructed side	NA	NA
Zong et al. 2019 [22]	20	Cervical cancer (FIGO I)	Clear cell adenocarcinoma	NR	NR	NR
	27	Cervical cancer (FIGO IIB)	Adenocarcinoma	NR	NR	NR
	31	Cervical cancer (FIGO IIA)	Clear cell adenocarcinoma	NR	NR	NR
	38	Cervical cancer (FIGO IIA)	Adenocarcinoma	NR	NR	NR
Lei and Zhang et al. 2024 [23]	40	Vaginal cancer (FIGO I)	Clear cell adenocarcinoma	Obstructed side	16+	No
Grant and Pierce et al. 1952 [24]	35	Cervical cancer (FIGO IIA)	Adenocarcinoma	Non-obstructed side	NR	NR
Zeeshan-ud-din et al. 2009 [25]	27	Vaginal cancer (FIGO III)	Clear cell adenocarcinoma	Obstructed side	NR	No
Uehara et al. 2010 [26]	54	Vaginal cancer (FIGO I)	Clear cell adenocarcinoma	Obstructed side	NR	No
Zeng et al. 2023 [27]	NR (“middle-aged”)	Endometrial cancer (FIGO 2009 IIIC1)	Serous adenocarcinoma	NR	NA	NA
Parisi et al. 2026	22	Borderline Ovarian Tumor (FIGO IIIA2)	Serous borderline ovarian tumor with focal microinvasion and non-invasive peritoneal implants	Bilateral (ovary)	NA	NA

Abbreviations: DES = Diethylstilbestrol; FIGO = International Federation of Gynaecology and Obstetrics; NA = not applicable; NR = not reported.

**Table 2:** Obstetric history, treatment strategies, and oncologic outcomes.

Author	Gravida/Para	Treatment	Follow-Up (Months)	Outcome
Mabuchi et al. 2022 [12]	0 G	RH, total vaginectomy, BSO, PLND	3	NED
Watanabe et al. 2012 [13]	2 G 2 P	CHRT, APE, total vaginectomy	NR	NR
	2 G 2 P	RH	NR	NR
Kaba et al. 2013 [14]	2 G 2 P	RH, BSO, PLND, PALND, omentectomy, proximal vaginectomy	15	NED
Cordoba et al. 2017 [15]	2 G 2 P	PALND + concomitant CHRT	30	NED
Kusunoki et al. 2018 [16]	3 G 2 P	RH, CHRT	12	NED
Oka et al. 2020 [17]	0 G	RH, CHRT	24	NED
Mei et al. 2020 [18]	1 G 1 P	RH, BSO, PLND, CHRT	17	NED
Tanase et al. 2021 [19]	0 G	RH, BSO, PLND	12	NED
Almulhim et al. 2021 [20]	0 G	Cystectomy	NR	NR
Kobayashi et al. 2021 [21]	2 G 1 P	RH, BSO, PLND, CHT	6	NED
Zong et al. 2019 [22]	NR	RH, BSO, PLND, total vaginectomy	36	NED
	NR	RH, BSO, +total vaginectomy, CHT	24	DOD Local recurrence and kidney failure after 1 year
	NR	RH, BSO, PLND, PALND, CHRT	18	DOD Local recurrence and distant metastases after 4 months
	NR	RH, BSO, PLND, PALND, RT	24	DOD Distant metastases after 1 year
Lei and Zhang et al. 2024 [23]	2 G 1 P	TH, tumor resection, CHT	60	NED Distant metastases after 1 year
Grant and Pierce et al. 1952 [24]	0 G	TH, PLND	NR	NR
Zeeshan-ud-din et al. 2009 [25]	NR	TH, partial vaginectomy, PLND	NR	NR
Uehara et al. 2010 [26]	4 G 3 P	APE	42	NED
Zeng et al. 2023 [27]	NR	TH, BSO, PLND, PALND, omentectomy, CHRT	6	NED
Parisi et al. 2026	0 G (Not attempted)	Cystectomy, infracolic omentectomy, peritoneal staging and washing	6	NED

Abbreviations: APE = anterior pelvic exenteration; BSO = bilateral salpingo-oophorectomy; CHRT = chemoradiotherapy; CHT = chemotherapy; DOD = dead of disease; G = gravida; NED = no evidence of disease; NR = not reported; P = para; PALND = para-aortic lymph node dissection; PLND = pelvic lymph node dissection; RH = radical hysterectomy; RT = radiotherapy; TH = total hysterectomy.

## Data Availability

No new data were created or analyzed in this study.

## Supplementary Materials

The following supporting information can be downloaded at: https://www.mdpi.com/article/10.3390/jcm15082824/s1, Table S1: PRISMA 2020 Checklist.

## Abbreviations

The following abbreviations are used in this manuscript:

BOT	Borderline ovarian tumor
DES	Diethylstilbestrol
FSS	Fertility-sparing surgery
HWWS	Herlyn–Werner–Wunderlich syndrome
OHVIRA	Obstructed hemivagina and ipsilateral renal anomaly
